# Assessment of Pediatric Nurses' Knowledge Regarding Pain Management in Children in Public Hospitals in Greece

**DOI:** 10.7759/cureus.105835

**Published:** 2026-03-25

**Authors:** Eleni N Albani, Eleni Strakantouna, Spyridon Rigatos, Constantinos Togas, Chrysanthi Sotiriadou, Konstantinos T Petsios, Anastasios Tzenalis

**Affiliations:** 1 Nursing, University of Patras, Patras, GRC; 2 Science and Technology, Hellenic Open University, Athens, GRC; 3 Oncology Nursing, Papageorgiou General Hospital, Thessaloniki, GRC; 4 Nursing, National and Kapodistrian University of Athens, Athens, GRC

**Keywords:** knowledge, nurses, pain assessment, pain management, pediatric nursing

## Abstract

Purpose: This study aimed to assess pediatric nurses' knowledge and skills in pain management and examine the factors influencing their proficiency in this area.

Design and methods: A cross-sectional study was carried out to assess the knowledge of pediatric nurses in Greece. Data were collected from six public pediatric hospitals using a convenience sampling method, yielding a sample of 154 nurses. The Demographic Information Questionnaire and the Knowledge and Attitudes Survey Regarding Pain (KASRP) were utilized. Statistical analysis was performed using Statistical Product and Service Solutions (SPSS) software.

Results: The study found that pediatric nurses had a mean score of 51.98% on the pain management knowledge questionnaire, highlighting significant gaps in understanding, particularly regarding opioid use and pain intensity indicators. Scenario-based decision-making revealed that only a small percentage of participants correctly identified the appropriate dosage for pediatric pain management. Key areas for improvement were identified, especially in the proper use of opioids and pain assessment scales.

Discussion: The findings of this study highlight the need for targeted educational interventions and training programs to enhance pediatric nurses' competencies in pain management. Improving nurses' knowledge and clinical decision-making could contribute to more effective pain relief and higher-quality care for pediatric patients.

## Introduction

Pain is a personal and subjective experience, unique to each individual. It is defined as "an unpleasant sensory and emotional experience associated with actual or potential tissue damage" [1]. It is a complicated experience that presents numerous challenges in both diagnosis and treatment for healthcare providers. Additionally, chronic pain, characterized as pain that lasts or recurs for over three months, is a condition that greatly diminishes the quality of life in both adults and children [1].

The American Society of Anesthesiologists Task Force on Acute Pain Management found that, in infants and children, emotional factors play a major role in pain. Things such as being away from parents, unfamiliar surroundings, and feeling insecure can intensify pain - sometimes even more than the physical pain from surgery itself [2].

It is estimated that approximately 79% of hospitalized patients experience pain at some point during their stay, and 55%-78.6% of inpatients experience moderate-to-severe pain [3,4]. In addition, it is estimated that approximately 38% of children and adolescents worldwide suffer from chronic pain [5], and children are regarded as a vulnerable group when it comes to unmanaged pain, with pain relief being directly proportional to age [6].

The causes of pediatric pain include chronic diseases and conditions (e.g., cancer, anemia, diabetes, and inflammatory bowel disease), injuries, trauma, burns, and invasive procedures during the therapy course [7].

Chronic pain in children and adolescents can reduce school attendance, academic performance, social participation, and physical activity while increasing anxiety and depression and lowering overall quality of life [8,9]. Untreated pain in children can cause long-term physical, emotional, and developmental issues, increase the risk of chronic pain, and lead to longer hospital stays, higher healthcare costs, and more readmissions [10].

The importance of optimal pediatric pain management is widely recognized [11], and effective pain management is a cornerstone of quality healthcare in pediatric nursing [12]. Moreover, access to appropriate pain relief has been declared as an ethical obligation and a human right [13].

To ensure standardized nursing care, guidelines have been established to assess pain as the fifth vital sign [14]. Nevertheless, including pain management within goals, rights, and policies does not ensure its implementation, as pain remains primarily a subject of research in nursing [10].

The World Health Organization recommends using multimodal pain management in children, combining medication with non-drug methods. Non-pharmacological strategies can include sucking (nutritive or non-nutritive) and distraction techniques, such as play, storytelling, music, virtual reality, cuddling, and similar activities [15].

Nurses dedicate more time and play an active role in managing pain, as they are responsible for assessing pain, administering pharmacological treatments, independently applying non-pharmacological methods, reassessing the patient's condition, and involving the family in the process [13,16].

In pediatric and adolescent healthcare, pain is evaluated and re-evaluated using validated scales that are chosen based on the individual's developmental stage and cognitive abilities. Research suggests that pediatric nurses often struggle to select appropriate pain assessment (PA) tools, leading to suboptimal pain management [17]. The assessment tools include the Neonatal Infant Pain Scale (NIPS) for newborns up to two months old; the Wong-Baker FACES Pain Rating Scale for children older than three years; the Face, Legs, Activity, Cry, and Consolability (FLACC) scale for children between two months and seven years old; the Numerical Verbal Scale (NVS), considered the gold standard, which relies on self-reports from children over seven years old; and the Comfort-Behavior Scale (COMFORT-b) for children who are under continuous sedation [15].

Despite advancements in pain management protocols and the development of standardized assessment tools, there are significant gaps in knowledge among healthcare professionals, particularly nurses [18]. These deficiencies are often linked to insufficient training, misconceptions about analgesics, and limited adoption of evidence-based practices [19]. Moreover, certain factors, such as guidelines, protocols, staff knowledge, pain location, and pain suppression, have been found to interfere with proper pain assessment [20,21].

Research by Pretorius et al. found that many healthcare providers lack confidence in using opioids, often opting for non-opioid analgesics even in situations where opioids are indicated [22]. This reluctance to administer opioids can significantly impair the quality of pain relief for pediatric patients, especially those undergoing surgery, cancer treatments, or palliative care [23]. Additionally, nurses' personal beliefs and attitudes toward pain management can influence their clinical decision-making, contributing to disparities in pediatric pain treatment [24].

Miftah et al. found that most nurses are knowledgeable and follow protocols for pediatric pain management, using both pharmacological and non-pharmacological methods, though anesthesia can sometimes complicate PA [25].

Fenta et al. reported that most nurses in Ethiopia (74.7%) had inadequate knowledge and attitudes toward pediatric pain management. Greater pediatric nursing experience and formal pain management training were linked to higher knowledge and attitude scores [13].

In general, a meta-analysis conducted by McCabe et al. revealed that 45.59% of the 1,090 participants possessed sufficient or higher levels of knowledge regarding acute pain management. This indicates that, overall, patients have typically been provided with less than optimal care [26].

Education and clinical experience improve nurses' competency in pain management: formal training enhances assessment and medication skills, while personal experiences with pain boost empathy and understanding of patient needs [27,28]. Education can boost nurses' knowledge, attitudes, and practices in pain management, but its effects may fade over time, highlighting the need for ongoing training [29]. Pediatric nurses should receive continual education and skill development to maintain and improve care quality [30].

While some studies show that educational interventions on pain knowledge, assessment, and management can be effective, this is not consistent across all research. There is significant criticism regarding the quality and comparability of these studies, with recommendations for improvements and standardization in their design and interventions [23].

Achaliwie et al. found that educational programs can improve nurses' knowledge, attitudes, skills, and practices in pain management, though some misunderstandings - particularly about pharmacological methods - may remain [29].

In Greece, most nurses hold a university-level education, while nurse assistants graduate from higher vocational training institutes (SAEK) or vocational high schools (EPAL). EPAL combines general education with vocational training, and its graduates must complete a six-month internship in healthcare facilities to obtain a license. University nursing students, on the other hand, fulfill their clinical internship during the eighth semester as required by the official curriculum. Legislation clearly defines the roles and duties of nurses and nurse assistants. Medication administration is reserved for university-educated nurses, though nurse assistants may do so in emergencies under supervision.

Aiming to identify knowledge gaps and inform targeted educational and policy interventions to improve pediatric care, this study evaluated Greek pediatric nurses' knowledge, skills, and decision-making in pain management, with a particular emphasis on the clinical use of opioids. Specifically, it examined the following: (1) What is the extent of nurses' knowledge of pain management? (2) How do nurses' demographic characteristics influence their knowledge? (3) How do their professional experience and continuing education affect their knowledge? (4) How do personal pain experiences influence their knowledge?

## Materials and methods

Study design

This study utilized a cross-sectional design to evaluate the knowledge and decision-making practices of pediatric nurses concerning pain management in Greece. Participants were recruited from various pediatric hospitals across the country. The hospitals involved in the study were General Hospital Papageorgiou, Karamandaneio Prefecture Children Hospital of Patras, General University Hospital of Patras, General Hospital of Athens "G. Gennimatas", Children's Hospital "P. & A. Kyriakou", and Penteli Children's General Hospital.

Sampling and participants

The sample included 154 pediatric nurses working in six public pediatric hospitals across Greece. Participants who were actively employed in a pediatric unit were recruited using a convenience sampling method. Participants were excluded if they did not work in a pediatric unit.

Measures

A composite questionnaire was used, which included demographic and work-related information, as well as the Greek version of the Knowledge and Attitudes Survey Regarding Pain (KASRP) [31-33].

Demographic and Work-Related Information/Personal Experience of Pain

Demographic characteristics collected included gender, age, educational level, work area, postgraduate education in pain management, and their knowledge and beliefs with personal experience of acute or chronic pain. In addition, nurses were asked questions regarding job satisfaction, such as whether they would change their workplace or profession if given the opportunity. Finally, they were asked to indicate whether they personally experienced acute or chronic pain.

Knowledge and Attitudes Survey Regarding Pain (KASRP)

The KASRP tool was first created by Ferrell and McCaffery in 1987 and has been extensively utilized ever since. The items within the tool, which assess knowledge and attitudes toward pain, are not separated but measured together due to their strong interrelation. Apart from the questions, this scale includes two patient case studies that require decisions regarding PA and pharmacological management. The authors slightly modified these scenarios to adapt them for children rather than adults (Appendix 1). The revised version of the KASRP tool has demonstrated high test-retest reliability and internal consistency, with Pearson's correlation coefficient of r > 0.80 and Cronbach's alpha coefficient of > 0.70 [31,32]. The test-retest reliability and internal consistency of the Greek version used in this study were also found to be adequate, with Pearson's correlation coefficient of r = 0.69 and Cronbach's alpha coefficient of 0.88 [33]. The updated 1997 KASRP tool includes 39 questions: 21 true/false, 14 multiple-choice, and 2 case studies. Each case study has two parts - one multiple-choice question and one where participants assess pain using a 0-10 scale - focusing on decisions related to pain and medication. Correct responses can be scored for the total number of items, individual items, or specific groups of items. Tapp and Kropp modified the KASRP tool to focus on postoperative pain by removing eight questions related to children's pain and cancer pain [34]. The modified version, known as the KASRP-P, contains 31 items. The subscales identified by Tapp and Kropp include PA with seven items, general pain management (GPM) with nine items, and the use of analgesics (UoA) with 15 items.

Procedure

Participants were approached in their workplace, and the questionnaires were distributed by their supervisors following coordination with members of the research team. The questionnaires were provided in printed form and could be completed at the participants' convenience. Completed questionnaires were returned in the subsequent days. The data collection process required approximately 10-15 minutes per participant and ensured both anonymity and confidentiality. Data collection was conducted over a six-month period, from January to June 2023. No incentives or rewards were offered for participation.

Statistical analysis

The data were examined using descriptive and inferential statistical methods. Continuous variables were presented using means, standard deviations, and ranges, while categorical variables were shown as frequencies and percentages. Group differences were evaluated with independent samples t-tests, and effect sizes were determined using Cohen's d. Relationships between continuous variables were measured using Pearson's correlation coefficient (r). All analyses were conducted with Statistical Product and Service Solutions (SPSS, version 29; IBM SPSS Statistics for Windows, Armonk, NY), and a p-value of less than 0.05 was considered statistically significant.

Ethical consideration

The study was approved by the scientific councils or ethics committees of all participating pediatric hospitals (approval number: 316/13-09-2022). It was conducted in accordance with national and international regulations governing research involving human subjects. The protection of personal data complied with the General Data Protection Regulation (GDPR) and the provisions of national law 4624/2019. Informed consent was obtained prior to the administration of the questionnaires. Participation was voluntary, with no obligation to take part. Confidentiality was strictly maintained, and access to data was restricted to the research team. Study findings will be made available to participants upon request. Before completing the questionnaires, participants signed a consent form confirming their informed agreement to participate and acknowledgment of the study's objectives. They were also made aware that they could leave the study at any point without facing any negative repercussions.

## Results

Out of 180 questionnaires distributed, 154 were filled out and returned, resulting in a response rate of 85.60%.

The mean age of the participants was 43 years (N = 154, M = 43.18, SD = 8.89, min = 19, max = 59, range = 40). Participants had an average of 18.5 years of experience (M = 18.69, SD = 9.60, min = 1, max = 38, range = 37). The remaining demographic and work-related characteristics of the sample, as well as pain experience, are presented in Table 1.

**Table 1:** Demographic and work-related characteristics of the sample and pain experience

Variables	Frequency	Percent
Gender		
Male	18	11.69
Female	136	88.31
Marital/Relationship Status		
Married	106	68.83
Single	36	23.38
Divorced	8	5.19
Widowed	3	1.95
Other	1	0.65
Are you a parent?		
Yes	106	68.83
No	48	31.17
Hospital		
General Hospital Papageorgiou	55	35.71
Karamandaneio Prefecture Children Hospital of Patras	34	22.08
General University Hospital of Patras	19	12.34
General Hospital of Athens "G. Gennimatas	17	11.04
Children's Hospital "P. & A. Kyriakou"	17	11.04
Penteli Children's General Hospital	12	7.79
Nursing Education		
High School/Technical Vocational Schools/Vocational Training Institute	26	16.88
Technological Educational Institute	109	70.78
University	19	12.34
Postgraduate Education		
Pediatric Nursing Specialty	23	14.94
Postgraduate Degree	56	36.36
Employment Status		
Permanent	120	77.92
Contract	29	18.83
Other	5	3.25
Participation in a training program/conference/seminar on pain management?		
Yes	44	28.57
No	110	71.43
If given the opportunity, would you change your workplace?		
Yes	72	46.75
No	82	53.25
If given the opportunity, would you change your profession?		
Yes	79	51.30
No	73	47.40
Do you personally experience acute or chronic pain?		
Yes	61	39.61
No	93	60.39

Assessment of knowledge and attitudes regarding pain

The mean percentage of correct answers to the questionnaire items was 51.98% (possible range: 0-100) (N = 154, M = 51.98, min = 11.58, max = 80.65, range = 58.06). A total of 54.55% of participants (N = 84) answered more than half of the questions correctly, while 45.9% (N = 70) answered less than half correctly.

Percentage of correct answers per question

The percentages of correct answers in the KASRP are presented in Table 2.

**Table 2:** Assessment of knowledge regarding pain (part 1) Assessment of knowledge regarding pain: false and correct answers Sources: The Knowledge and Attitudes Survey Regarding Pain (KASRP) and its validated Greek version [31-33] were utilized with written permission obtained from the original authors.

Item	Correct Answer	Frequency and Percentage of Correct Answers
1. Changes in a patient’s vital signs can be used as reliable indicators of pain intensity.	False	28 (18.18%)
2. Children under the age of two have reduced sensitivity to pain and limited memory of painful experiences due to their undeveloped nervous systems.	False	82 (53.25%)
3. If a patient’s attention can be diverted from the pain, it might indicate that they do NOT have severe pain.	False	87 (57.79%)
4. Patients can sleep despite experiencing severe pain.	False	110 (71.43%)
5. Similar stimuli in different children/patients produce pain of the same intensity.	False	111 (72.08%)
6. Nonsteroidal anti-inflammatory drugs (NSAIDs) are NOT effective analgesics for painful bone metastases.	False	76 (49.35%)
7. Non-pharmacological interventions (e.g., heat, music, dim lighting, cuddling) are very effective in controlling mild-to-moderate pain but are rarely useful for very severe pain.	True	130 (84.42%)
8. Respiratory depression rarely occurs in patients receiving steady opioid doses over a period of months.	True	81 (52.60%)
9. Combining analgesics that work via different mechanisms (e.g., combining NSAIDs with an opioid) can lead to better pain control with fewer side effects compared to using a single analgesic.	True	132 (85.71%)
10. The usual duration of analgesia for intramuscular meperidine (pethidine) is 4-5 hours.	True	55 (35.71%)
11. Research has shown that promethazine (Phenergan) is a reliable potentiator of opioid analgesics.	False	61 (39.61%)
12. Patients with a history of substance abuse should not receive opioids for pain due to a high risk of addiction.	False	64 (41.56%)
13. Beyond a certain dose of opioids, increasing the dose will not improve pain relief.	False	73 (47.40%)
14. Patients in pain should be encouraged to tolerate as much pain as possible before using a pain relief method.	False	91 (59.09%)
15. Children under the age of 11 cannot reliably report pain, so the nurse should rely on the parent’s assessment of the child’s pain intensity.	False	91 (59.09%)
16. Some patients’ spiritual beliefs may lead them to view pain as necessary.	True	108 (70.13%)
17. After the initial recommended dose of an analgesic, subsequent doses should be adjusted based on the patient’s response to analgesia.	True	129 (83.77%)
18. Patients should be advised to use non-pharmacological techniques alone and not alongside analgesics.	False	107 (69.48%)
19. Administering sterile water to patients (placebo) is often a useful test to determine if the pain is real.	False	38 (24.68%)
20. For cold and heat therapies to be effective, they should only be applied to the painful area.	True	84 (54.55%)
21. If the source of a patient’s pain is unknown, opioids should not be used during the pain episode; a period of evaluation should be allowed, as administering analgesia might mask the ability to correctly diagnose the cause of the pain.	False	25 (16.23%)

The highest percentage of correct answers was recorded for the following items:

Item 9: “Combining analgesics that work via different mechanisms (e.g., combining nonsteroidal anti-inflammatory drugs (NSAIDs) with an opioid) can lead to better pain control with fewer side effects compared to using a single analgesic” (N = 132, 85.71%).

Item 7: “Non-pharmacological interventions (e.g., heat, music, dim lighting, cuddling, etc.) are very effective in controlling mild-to-moderate pain but are rarely useful for very severe pain” (N = 130, 85.42%).

Item 17: “After the initial recommended dose of an analgesic, subsequent doses should be adjusted based on the patient's response to analgesia” (N = 129, 83.77%).

In contrast, the lowest percentage of correct answers was recorded for the following items:

Item 21: “If the source of a patient's pain is unknown, opioids should not be used during the pain episode; a period of evaluation should be allowed, as administering analgesia might mask the ability to correctly diagnose the cause of the pain” (N = 25, 16.23%).

Item 1: “Changes in a patient's vital signs can be used as reliable indicators of pain intensity” (N = 28, 18.18%).

The percentage of correct answers for the remaining items is presented in Table 3.

**Table 3:** Assessment of knowledge regarding pain (part 2) Assessment of knowledge regarding pain: false and correct answers Sources: The Knowledge and Attitudes Survey Regarding Pain (KASRP) and its validated Greek version [31-33] were utilized with written permission obtained from the original authors.

Question	Answer Choices	Correct Answer	Percentage of Correct Answers
22. The recommended route for administering opioid analgesics to patients with persistent cancer-related pain is:	Intravenously, intramuscularly, subcutaneously, orally, I don’t know	Orally	10 (6.49%)
23. The recommended route for administering opioid analgesics to patients with sudden onset, severe pain such as trauma or postoperative pain is:	Intravenously, intramuscularly, subcutaneously, orally, rectally, I don’t know	Intravenously	84 (54.55%)
24. Which of the following analgesic medications is considered the drug of choice for treating prolonged moderate to severe pain in cancer patients?	Brompton’s cocktail, codeine, morphine, pethidine, I don’t know	Morphine	70 (45.45%)
25. Which of the following IV doses of morphine administered over a 4-hour period is equivalent to 30 mg oral morphine given every 4 hours?	Morphine 5mg, Morphine 10mg, Morphine 30mg, Morphine 60mg	Morphine 10 mg	50 (32.45%)
26. Analgesics for postoperative pain should initially be administered:	1) 24 hours on a fixed schedule. 2) Only when the patient requests the medication. 3) Only when the nurse assesses that the patient has moderate or greater discomfort	24 hours on a fixed schedule	130 (84.42%)
27. A patient with persistent cancer pain has been taking opioid analgesics daily for two months. Yesterday, the patient received morphine 200 mg/hour intravenously. Today, the patient is receiving 250 mg/hour intravenously. The likelihood of clinically significant respiratory depression in the patient is:	1) Less than 1%. 2) 1-10%. 3) 11-20%. 4) 21-40%. 5) >41%	Less than 1%	46 (29.87%)
28. The most likely reason a patient with pain would request increased doses of pain medication is:	1) The patient is experiencing increased pain. 2) The patient is experiencing increased anxiety. 3) The patient asks for more attention from the staff	The patient is experiencing increased pain	119 (77.27%)
29. Analgesia for cancer pain should be administered:	1) At specific times. 2) Only when the patient feels pain. 3) Only when the nurse determines that the patient has moderate or greater discomfort	At specific times	112 (72.73%)
30. The most accurate assessment of the patient’s pain intensity is made by:	1) The attending physician. 2) The patient’s nurse. 3) The patient. 4) The pharmacist. 5) The patient’s family	From the patient	47 (30.52%)
31. Which of the following describes the best approach to cultural factors in the care of patients with pain?	1) There are no longer cultural influences. 2) Nurses should use their knowledge. 3) Patients should be assessed individually	Patients should be assessed individually	112 (72.73%)

The highest percentage of correct answers (N = 130, 84.42%) was for the item: "Analgesics for postoperative pain should initially be administered…" (correct answer: 24 hours on a fixed schedule). In contrast, the lowest percentage of correct answers (N = 10, 6.49%) was for the item: "The recommended route for administering opioid analgesics to patients with persistent cancer-related pain is…" (correct answer: orally).

In response to the question, “What do you think is the percentage of patients who exaggerate their pain intensity?”, the following answers were recorded in Table 4.

**Table 4:** Responses to the question “What do you think is the percentage of patients who exaggerate their pain intensity?”

What do you think is the percentage of patients who exaggerate their pain intensity?	Frequency	Percent
10%	19	12.34
20%	41	26.62
30%	29	18.83
40%	13	8.44
50%	16	10.39
60%	15	9.74
70%	15	9.74
80%	7	4.55

Next, differences in correct answer scores based on the demographic and work-related characteristics of the sample were examined (Table 5).

**Table 5:** Differences in correct answer scores based on the demographic and work-related characteristics of the sample

Items	N	Mean	Std. deviation	Std. error Mean	t	df	Two-sided p
Postgraduate education							
Pediatric nursing specialty	23	48.95	8.06	1.6	-3.02	79	0.003
Postgraduate degree	56	56.4	10.68	1.4			
Participation in a training program/conference/seminar on pain management							
Yes	44	55.13	9.45	1.41	2.45	152	0.016
No	110	50.72	10.49	0.99			
If given the opportunity, would you change your workplace?							
Yes	72	54.19	11.31	1.32	2.56	152	0.012
No	82	50.02	9.07	0.1			
Do you personally experience acute or chronic pain?							
Yes	61	54.95	11.22	1.44	2.85	152	0.005
No	93	50.17	9.46	0.98			

Significantly higher correct answer scores were observed among the following groups: (1) Participants with a postgraduate degree compared to those with a pediatric nursing specialty (t = -3.02, df = 79, p = 0.003, Cohen's d = -0.74); (2) participants who had attended a training program, conference, or seminar on pain management compared to those who had not (t = 2.45, df = 152, p = 0.016, Cohen's d = 0.43); (3) participants who stated they would change their workplace if given the opportunity compared to those who stated they would not change their workplace (t = 2.56, df = 152, p =0.012, Cohen's d = 0.41); and (4) participants who personally experienced acute or chronic pain compared to those who did not experience such pain (t = 2.85, df = 152, p = 0.005, Cohen's d = 0.47).

The correlations are presented in Table 6. 

**Table 6:** Correlations between age, years of experience, and the score for correct answers on pain knowledge Note **: Correlation is significant at the 0.01 level (2-tailed).

Variables		Age	Years of Experience	Score of Correct Answers on Pain Knowledge
Age	Pearson's Correlation	-	0.875^**^	0.080
	Sig. (2-tailed)	-	<0.001	0.361
	N	-	154	154
Years of experience	Pearson's Correlation	0.875^**^	-	0.015
	Sig. (2-tailed)	<0.001	-	0.158
	N	154	-	154
Score of correct answers on pain knowledge	Pearson Correlation	0.080	0.115	-
	Sig. (2-tailed)	0.361	0.158	-
	N	154	154	-

No significant correlation was found between age and the score of correct answers on pain knowledge (r = 0.080, p = 0.361). Similarly, no significant correlation was found between years of experience and the score of correct answers on pain knowledge (r = 0.115, p = 0.158).

Scenario results

Two case studies of patients were presented, requiring decisions regarding PA and pharmacological management. The full scenarios (C1a & C1b) are available in the supplementary material (Appendix 1).

In Scenario C1a, where the correct answer was “Tramal 3 mg IV will be given now,” only 7.64% of participants (11 out of 144 valid responses) selected the correct option. The majority of participants (N = 78, 54.2%) chose “Do not administer Tramal at this time,” while 26.4% (N = 38) selected “Tramal 1 mg IV will be given now,” and 11.8% (N = 17) selected “Tramal 2 mg IV will be given now."

In Scenario C1b, the correct answer was again “Tramal 3 mg IV will be given now.” A slightly higher percentage of participants (13%, or 19 out of 146 valid responses) selected the correct option. However, the majority (N = 70, 47.95%) chose “Tramal 1 mg IV will be given now,” followed by 19.86% (N = 29) who selected “Do not administer Tramal at this time,” and 19.18% (N = 28) who chose “Tramal 2 mg IV will be given now.”

## Discussion

The findings of this study highlight significant gaps in the knowledge of pediatric nurses in Greece regarding pain management, which align with previous international research. Specifically, the mean percentage of correct answers (51.98%) reflects a moderate level of understanding, with only 54.1% of participants answering more than half of the questions correctly. These results are consistent with similar studies in other countries, where nurses have demonstrated inadequate knowledge of pain management. For instance, Elcigil et al. found that nurses in Turkey scored an average of 53% on a pain management knowledge test, while Kizza et al. reported that Ugandan nurses exhibited significant deficits in opioid use knowledge for pediatric pain relief [35,36].

Regarding areas of strength, participants demonstrated a solid understanding of the benefits of multimodal analgesia, with 84.5% correctly identifying the effectiveness of combining analgesics for improved pain control. Additionally, the vast majority recognized the efficacy of non-pharmacological interventions, findings that align with those of Stanley et al. [20], who reported that nurses recognize the importance of integrating both pharmacological and non-pharmacological strategies in pain management.

However, critical gaps were identified in key areas, particularly in the use of opioids and the assessment of pain intensity. Only 16.23% of nurses correctly answered questions about opioid administration when the pain source is unknown, reflecting a widespread misconception that opioids should be avoided in such cases. Similarly, only 18.18% recognized that vital signs alone are not reliable indicators of pain intensity, a finding consistent with Lui et al., who reported that nurses often overestimate the risks associated with opioid use, leading to inadequate pain relief for pediatric patients [37].

Furthermore, misconceptions regarding opioid safety and addiction remain significant barriers to effective pain management. Pretorius et al. found that many healthcare providers exhibit reluctance in prescribing opioids, preferring non-opioid analgesics even in cases where opioid use is indicated [22]. The findings of this study support this trend, reinforcing concerns that opioid-related fears contribute to the undertreatment of pain in pediatric patients.

The findings also indicate that education is essential in influencing nurses' understanding of pain management. Studies indicate that nurses with specialized pain education demonstrate higher competency in PA and medication administration [27]. However, in this study, a lack of formal pain education was reported by a significant proportion of participants, which may have contributed to knowledge deficits. Given these findings, structured educational programs and policy initiatives are needed to enhance pediatric nurses’ understanding and implementation of evidence-based pain management practices [38].

These results highlight the urgent need for targeted interventions to address gaps in pain management knowledge among pediatric nurses in Greece, particularly regarding opioid use and the application of PA tools. Future studies should explore the impact of educational interventions on enhancing pediatric pain management practices and improving patient outcomes.

Scenario-based decision-making

In both clinical scenarios presented in this study, the majority of participants failed to select the correct dosage of Tramal (3 mg IV), with only 7.64% and 13% of participants responding correctly in Scenarios C1a and C1b, respectively. These findings align with Yava et al., who identified significant difficulties among nurses in accurately dosing opioids, often influenced by fears of over-sedation or addiction [39]. The frequent selection of incorrect options, such as "Do not administer Tramal at this time," suggests hesitancy and a lack of confidence in decision-making.

Factors influencing knowledge

Participants with postgraduate degrees, those who had attended pain management training, and those with personal experiences of pain demonstrated higher knowledge scores. These findings align with research by Al-Shaer et al., which highlights that continuous education and training significantly enhance nurses' competencies in pain management [40].

Limitations

This research has several limitations. Firstly, it depended on self-reported questionnaires, which could be influenced by response bias. Secondly, the use of convenience sampling restricts the ability to generalize the results. Furthermore, as the study was conducted exclusively in public pediatric hospitals in Greece, the results may not be generalizable to other healthcare settings, such as private pediatric clinics.

The results of this study emphasize the critical need for focused efforts to fill knowledge gaps and correct misunderstandings among pediatric nurses. Integrating comprehensive pain management training into nursing curricula and continuing professional development programs can enhance both theoretical understanding and clinical application. Notably, addressing fears and misconceptions about opioid use is essential, as research by Polit et al. underscores the negative impact of such concerns on effective pain management [41].

Future research should prioritize the development and evaluation of targeted educational interventions to improve pediatric nurses' knowledge of pain management. Longitudinal studies could assess the impact of structured pain management training on nurses' clinical decision-making and patient outcomes. Additionally, qualitative research exploring nurses' perceptions, barriers, and facilitators to effective pain management could provide deeper insights into the underlying causes of knowledge gaps and misconceptions. Comparative studies across various healthcare settings and countries may help identify best practices and inform standardized guidelines for pediatric pain management. Furthermore, investigating the integration of digital tools, such as mobile applications and virtual simulations, in PA training could offer innovative solutions to enhance nurses' competencies. Finally, future studies should examine the role of interprofessional collaboration in pediatric pain management, exploring how teamwork among nurses, physicians, and other healthcare professionals can optimize PA and treatment strategies.

## Conclusions

This study highlights significant gaps in pediatric nurses' knowledge of pain management in Greece, aligning with international research. While aspects such as multimodal pain management and non-pharmacological interventions were well understood, critical misconceptions persisted - particularly regarding opioid use and the reliability of PA methods. Higher knowledge scores were linked to postgraduate education, specialized training, and personal pain experiences, yet work experience alone did not correlate with improved understanding. Additionally, scenario-based decision-making revealed challenges in the practical application of pain management principles. These findings emphasize the need for targeted educational programs and ongoing professional training to address knowledge deficits, enhance clinical decision-making, and ultimately improve pediatric pain management outcomes.

## Appendices

Appendix 1

Case Studies

Scenario C1a: Two patient case studies are presented. For each patient, you are asked to make decisions regarding their pain management and pharmacological treatment.

Patient A: George is 15 years old, and this is his first day post-abdominal surgery. As you enter his room, he smiles at you and continues to talk and joke with his visitor. A vital signs evaluation reveals the following: BP = 110/70, HR = 90, Respiration = 20. On a scale from 0 to 10 (0 = no pain/discomfort, 10 = worst pain/discomfort), he rates his pain as 8.

Based on the patient’s report, mark the pain level on the scale below. Please circle the number that best represents your assessment of George’s pain.

           0           1            2               3               4              5                6              7               8            9             10

-----------------------------------------------------------------------------------------------------------------------

No pain/discomfort                                                                                                                       Unbearable pain/discomfort

This assessment was conducted two hours after administering 2 mg of IV Tramal. The patient experiences continuous pain every half hour, with a pain rating between 6 and 8, despite medication. No clinically significant sedation or other adverse effects have been observed. The patient considers a pain relief level of 2 to be tolerable. The physician's analgesia order states: "Tramal IV 1-3 mg every hour for pain relief."

Please circle the action you would take at this moment.

1. Do not administer Tramal at this moment.

2. Administer 1mg IV Tramal now.

3. Administer 2mg IV Tramal now.

4. Administer 3mg IV Tramal now.

Scenario C1b: Patient B: Robert is a 15-year-old boy on his first post-operative day following abdominal surgery. As you enter his room, he is lying quietly in bed, making unpleasant facial expressions when he moves. His vital signs are as follows: BP = 110/70, HR = 90, Respiration = 20. On a scale from 0 to 10 (0 = no pain/discomfort, 10 = worst pain/discomfort), he rates his pain as an 8.

Based on the patient's report, please circle the number on the scale below that best represents your assessment of Robert’s pain.

           0           1            2               3               4              5                6              7               8            9             10

-----------------------------------------------------------------------------------------------------------------------

No pain/discomfort                                                                                                                       Unbearable pain/discomfort

This assessment was conducted two hours after the administration of 2 mg IV Tramal. The patient reports continuous pain every half hour, with pain levels ranging between 6 and 8 despite medication. No clinically significant sedation or other adverse effects have been observed. The patient considers a pain relief level of 2 to be tolerable. The physician's analgesia order states: "Tramal IV 1-3 mg every 1 hour for pain relief."

Please circle the action you would take at this moment.

1. Do not administer Tramal at this moment.

2. Administer 1mg IV Tramal now.

3. Administer 2mg IV Tramal now.

4. Administer 3mg IV Tramal now.
